# Content-Based Histopathological Image Retrieval

**DOI:** 10.3390/s25051350

**Published:** 2025-02-22

**Authors:** Camilo Nuñez-Fernández, Humberto Farias, Mauricio Solar

**Affiliations:** 1 Departamento de Informática, Universidad Tecnica Federico Santa Maria, Campus San Joaquin, Santiago 8940897, Chile; camilo.nunezf@usm.cl; 2 Institute for Multidisciplinary Research, Universidad de La Serena, La Serena 8380453, Chile; hfarias@userena.cl

**Keywords:** content-based image retrieval, feature embedding, feature fusion, histopathological image, transfer learning

## Abstract

Feature descriptors in histopathological images are an important challenge for the implementation of Content-Based Image Retrieval (CBIR) systems, an essential tool to support pathologists. Deep learning models like Convolutional Neural Networks and Vision Transformers improve the extraction of these feature descriptors. These models typically generate embeddings by leveraging deeper single-scale linear layers or advanced pooling layers. However, these embeddings, by focusing on local spatial details at a single scale, miss out on the richer spatial context from earlier layers. This gap suggests the development of methods that incorporate multi-scale information to enhance the depth and utility of feature descriptors in histopathological image analysis. In this work, we propose the Local–Global Feature Fusion Embedding Model. This proposal is composed of three elements: (1) a pre-trained backbone for feature extraction from multi-scales, (2) a neck branch for local–global feature fusion, and (3) a Generalized Mean (GeM)-based pooling head for feature descriptors. Based on our experiments, the model’s neck and head were trained on ImageNet-1k and PanNuke datasets employing the Sub-center ArcFace loss and compared with the state-of-the-art Kimia Path24C dataset for histopathological image retrieval, achieving a Recall@1 of 99.40% for test patches.

## 1. Introduction

The increase in the amount of data produced by healthcare institutions [1], due to the improved accessibility and advances in the development of devices, poses several challenges for physicians to diagnose in an accurate and fast way [2,3]. In the histopathology domain, these challenges could be interpreted as an analysis of various types of whole-slide images (WSIs) that can reach up to 100,000 × 100,000 pixels. In this scenario, tumors could be localized in a restricted zone of just a few hundred pixels [4,5]. For this reason, and with the rise in computer-aided approaches in computer vision, specialists have utilized multiple techniques to support their task. One such technique is Content-Based Image Retrieval (CBIR), which can assist in the fast and efficient analysis of medical images when compared to classical analysis. Given the large quantity of WSIs, the use of CBIR techniques in their analysis has become more frequent [6].

Content-Based Histopathological Image Retrieval (CBHIR) presents a more complex challenge compared to classical CBIR due to the high variability in the visual appearance components of cells in different tissues, including shape, color, or texture [7]. Nevertheless, deep learning models, such as Convolutional Neural Networks (CNNs) and Vision Transformers (ViTs), have contributed to the extraction of these components using pre-trained models [8] on a large dataset like ImageNet [9]. Although these methods can help as a feature extractor for CBHIR systems, they are not specialized enough to be used when the histopathological image domain changes due to the single modality these pre-trained models have [10]. For this reason, it is often necessary to retrain these models using the transfer learning strategy to specialize them in the domain of histopathological images. This could be highly cost in terms of time and computational resources, considering the large number of parameters these pre-trained models have and the large number of patches that public datasets usually have. Even if the specific-domain training is surpassed, the image descriptor is a single-scale embedding that only leverages the deeper single-scale linear layers or advanced pooling layers of the model, missing out on the richer spatial context from earlier layers that a multi-scale perspective of the image can offer.

To address the need for a multi-scale model for histopathological images, we introduce the Local–Global Feature Fusion Embedding Model (LGFFEM). The proposed model is compounded by a pre-trained backbone for multi-scale feature extraction, a trainable neck model for fusing local and global features, and a trainable pooling head for image descriptor embedding. The contributions of this proposal include the following:

- A unified model for extracting image descriptor embeddings from histopathological images using multi-scale local–global fused features trained with Sub-center ArcFace loss [11].
- Two novel fusion operations, called Local Aggregator and Global Aggregator, employing a channel attention mechanism to enhance local and global feature fusion.
- A validation of the proposed model using the state-of-the-art CBHIR dataset Kimia Patch24C [12], demonstrating improved Recall@1 through experiments with the proposed embeddings.

This article is structured as follows. Section 2 provides a summary of current research in CBHIR and multi-scale global–local fusion features. Section 3 introduces the proposal, and Section 4 details the complete methodology, experiments, and implementation results. Section 5 concludes with insights and outlines future work. The source code and the weights of the trained model are available from https://github.com/camilo-nunez/ffnir, accessed on 10 January 2025.

## 2. Related Works

The current design of CBHIR systems is based on deep learning models for the feature extraction process as an embedding of image descriptors, especially CNNs and ViTs. For example, ref. [4] proposes a tool to search for similar images named SMILY, based on a CNN ranking model that produces the embedding of image descriptors from a collection of tissue samples from TCGA (The Cancer Genome Atlas). The model used by SMILY was trained with a pair of multi-similarity losses using the distance among the embeddings of natural images (e.g., trains, animals, persons, etc.) [13].

Another proposal that uses CNN models trained with the multi-similarity loss is [14]. The authors used a mixed-attention mechanism with spatial attention and channel attention. The model was trained using a self-established histopathological image retrieval dataset and the public Kimia Path24C dataset [12]. In this project, the authors specify that the embedding of the image descriptor was extracted using bottleneck operations composed of squeeze-and-excitation blocks [15] and fully-connected layers.

Another approach with CNNs is proposed in [16], where the authors used a Convolutional Auto-Encoder with unsupervised training. Their proposed model reconstructs the input image and extracts the image descriptor embedding from the bottleneck of the auto-encoder.

The model proposed in [17] uses a novel Siamese CNN hashing model to avoid imbalanced classes and limited samples in histopathological image datasets. This Siamese model uses two pre-trained models with shared weights as feature extractors and a hash-code-generator layer. It was trained using a contrastive pair loss function and the public datasets Kather [18] and BreakHis [19].

A similar approach to a Siamese CNN as a feature extractor and trained with a contrastive loss is [20], where the embeddings of the image descriptor were extracted from the deeper layers using global average pooling. The Siamese model was trained for two specific histopathological image domains: skin cancer with a dataset of spitzoid melanocytic skin cancer provided by the University Clinic Hospital of Valencia, and breast cancer with the dataset BreakHis [19].

Within the paradigm of multi-scale and local–global feature descriptors, the authors of [10] presented a novel approach for the fusion of textural features extracted from a Global–Local Pyramid Pattern (GLPP) [21] and visual features derived from a CNN trained across multiple medical image domains such as X-ray, breast tumors, and skin lesions.

## 3. Proposed Method

### 3.1. Motivation

Based on most of the actual research on Content-Based Medical Image Retrieval (CBMIR) [22,23,24] and the works mentioned in the previous Section 2, the technique identified as common for feature extraction is a pre-trained CNN backbone. However, these models are biased with the classical datasets on which they were trained, and do not provide a proper specific-domain embedding for the histopathological image, as was mentioned in Section 1 [9,10]. Also, we note that these models do not exploit the correct use of the local and global features that can be provided by the backbone, whether these features are or are not fused as in [21]. Due to this, and based on the actual state-of-the-art image retrieval systems [25,26,27], we propose a novel Local–Global Feature Fusion Embedding Model (LGFFEM).

### 3.2. Local–Global Feature Fusion Embedding Model (LGFFEM)

**Figure 1:**
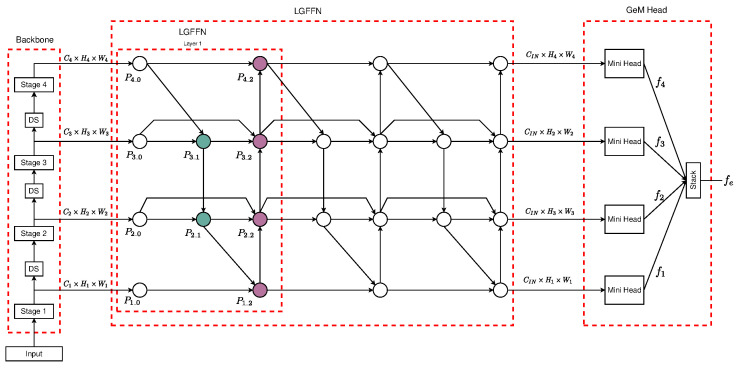
The LGFFEM architecture comprises a pre-trained backbone as a feature extractor from multi-scale stages, a trainable neck consisting of layers for local–global feature fusion, and a pooling head composed of trainable GeM mini-heads for each multi-scale fused feature from the neck.

The design of the LGFFEM is composed of three principal components: (I) a backbone that serves as a pre-trained network for feature extraction from multi-scale; (II) a trainable neck that serves as merge features from the multiple scale of the backbone and exploding a local–global fused technique; and (III) a Generalized Mean (GeM)-based head [28] that creates the image descriptor feature embedding, given the fused feature of the neck, using multiple mini-heads composed of trainable pooling layers, fully connected layers, and normalization operations. A comprehensive visualization of the final architecture is shown in Figure 1.

### 3.3. Feature Fusion Neck

#### 3.3.1. Feature Aggregator Units

Drawing parallels with ParseNet [29], the Feature Aggregator Units are designed to synergize both local and global features emanating from the backbone stages. Within the scope of this research, the distinctions between local and global features are articulated as follows:

1. Local Feature: The given features have a minimum receptive field; these features preserve complex spatial information, thus facilitating the generation of high-level features.
2. Global Feature: The features extracted from a generalization operation from an expansive receptive field are adept at capturing robust semantic information. These are categorized as low-level features.

##### Global Feature Aggregator

In the squeeze-and-excitation network study [15], to address the convolutional complexities, the squeeze-and-excitation block integrates global spatial information throughout the channels and subsequently consolidates these data through channel-wise dependencies. Then, for an input $X\in R^{C\times H\times W}$, the global feature operator of the squeeze-and-excitation block is defined as follows:

(1) $$G\left(X\right)=W_{2}\left(\delta \left(W_{1}\left(g\left(X\right)\right)\right)\right),$$

where both $W_{1}\in R^{\frac{C}{r}\times C}$ and $W_{2}\in R^{C\times \frac{C}{r}}$ represent learnable weights. The channel reduction factor is denoted as *r* and usually takes values within the set {2,4}. Here, δ is the activation function. The squeeze operator, symbolized as g(X), is formulated as a generalized spatial dependency operator, like a global average pooling mechanism.

Building upon the concept of point-wise channel representation introduced by the global feature operator, we focused on the interaction of the global spatial information throughout the channels. Guided by their insights, we chose to implement the point-wise convolution for cross-channel aggregation, specifically targeting the learnable weights $W_{1}$ and $W_{2}$. For the generalized spatial dependency operator, we used the Global Response Normalization layer, which increases the contrast and selectivity of channels [30]. Finally, a depthwise convolution was added in order to model the spatial relationship of the input.

As a result, the global feature operator formulated in our work can be expressed through the following equation:

(2) $$G\left(X\right)={\text{PW-Conv}}_{1\times 1}\left(\mathrm{GRN}(\delta \left({\text{PW-Conv}}_{1\times 1}({\text{DW-Conv}}_{3\times 3}(X)))\right)\right).$$

**Figure 2:**
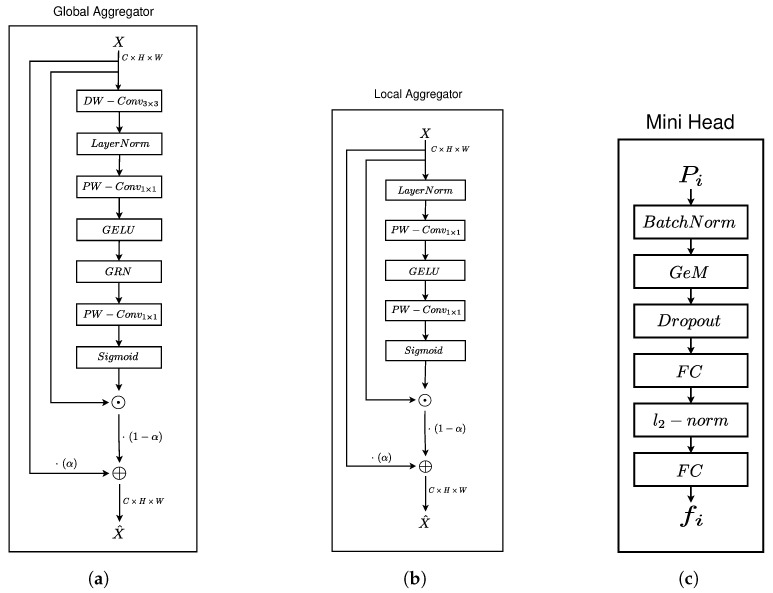
Illustration of the bottleneck operation for the Local and Global Aggregators and the pooling GeM mini-head. (**a**) Detailed schematic of the Global Feature Aggregator Unit. (**b**) Detailed schematic of the Local Feature Aggregator Unit. (**c**) Detailed schematic of the mini-head unit from the GeM head.

As a channel-wise attention, and similarly with squeeze-and-excitation networks, the novel Global Feature Aggregator is defined as

(3) $$\mathrm{GA}\left(X\right)=\alpha \cdot X⊕\left(1-\alpha \right)\cdot \left(X⊙\sigma \left(G(X)\right)\right),$$

where α is a trainable parameter, and σ denotes the Sigmoid function. A graphical representation of the Global Feature Aggregator is available in Figure 2a.

##### Local Feature Aggregator

Given the understanding that channel relationships shaped by convolution are inherently implicit and localized, the local feature operator functions as a simplified version of Equation (1). Notably, this operator excludes the generalized spatial dependency operator g(X):

(4) $$L\left(X\right)={\text{PW-Conv}}_{1\times 1}\left(\delta \left({\text{PW-Conv}}_{1\times 1}(X)\right)\right).$$

Finally, applying the channel-wise attention, the novel Local Feature Aggregator is defined as

(5) $$\mathrm{LA}\left(X\right)=\alpha \cdot X⊕\left(1-\alpha \right)\cdot \left(X⊙\sigma \left(L(X)\right)\right),$$

A graphical illustration of the Local Feature Aggregator can be found in Figure 2b.

#### 3.3.2. Neck’s Architecture

The schematic representation of this enhanced *neck*, denoted as Local–Global Feature Fusion Neck (LGFFN), is illustrated in Figure 1. The proposed architecture incorporates the node structure’s layer from the *BiFPN* [31] and the Fast Normalized Fusion weighting mechanism.

The input nodes of the neck are labeled as $P_{1\_0}$, $P_{2\_0}$, $P_{3\_0}$, and $P_{4\_0}$, each of which corresponds to the lateral output from the backend of the network. The intermediate nodes (the colors are consistent with the color scheme used to denote intermediate and terminal nodes, respectively, in Figure 1), namely $P_{3\_1}$ and $P_{2\_1}$, serve as internal aggregation fusion points. Similarly, the terminal nodes, denoted as $P_{1\_2}$, $P_{2\_2}$, $P_{3\_2}$, and $P_{4\_2}$, represent the outers aggregation fusion nodes and are analogous in function to their counterparts in the traditional BiFPN architecture layer.

The formal definitions for each of these feature fusion nodes are enumerated below:   

(6) $$\begin{array}{cc} P_{3\_1} & =\frac{w_{1}^{3\_1}\cdot \mathrm{GA}\left(P_{3\_0}\right)⊕w_{2}^{3\_1}\cdot \mathrm{LA}\left(\mathrm{Resize}\left(P_{4\_0}\right)\right)}{w_{1}^{3\_1}+w_{2}^{3\_1}+\epsilon }\\ P_{2\_1} & =\frac{w_{1}^{2\_1}\cdot \mathrm{GA}\left(P_{2\_0}\right)⊕w_{2}^{2\_1}\cdot \mathrm{LA}\left(\mathrm{Resize}\left(P_{3\_1}\right)\right)}{w_{1}^{2\_1}+w_{2}^{2\_1}+\epsilon }\\ P_{1\_2} & =\frac{w_{1}^{1\_2}\cdot \mathrm{GA}\left(P_{1\_0}\right)⊕w_{2}^{1\_2}\cdot \mathrm{LA}\left(\mathrm{Resize}\left(P_{2\_1}\right)\right)}{w_{1}^{1\_2}+w_{2}^{1\_2}+\epsilon }\\ P_{2\_2} & =\frac{w_{1}^{2\_2}\cdot \mathrm{LA}\left(P_{2\_0}\right)⊕w_{2}^{2\_2}\cdot \mathrm{LA}\left(P_{2\_1}\right)⊕w_{3}^{2\_2}\cdot \mathrm{GA}\left(\mathrm{Resize}\left(P_{1\_2}\right)\right)}{w_{1}^{2\_2}+w_{2}^{2\_2}+w_{3}^{2\_2}+\epsilon }\\ P_{3\_2} & =\frac{w_{1}^{3\_2}\cdot \mathrm{LA}\left(P_{3\_0}\right)⊕w_{2}^{3\_2}\cdot \mathrm{LA}(P_{3\_1})⊕w_{3}^{3\_2}\cdot \mathrm{GA}(\mathrm{Resize}\left(P_{2\_2}\right))}{w_{1}^{3\_2}+w_{2}^{3\_2}+w_{3}^{3\_2}+\epsilon }\\ P_{4\_2} & =\frac{w_{1}^{4\_2}\cdot \mathrm{LA}\left(P_{4\_0}\right)⊕w_{2}^{4\_2}\cdot \mathrm{GA}\left(\mathrm{Resize}\left(P_{3\_2}\right)\right)}{w_{1}^{4\_2}+w_{2}^{4\_2}+\epsilon } \end{array}$$

In the above equations, LA and GA represent both Local Feature Aggregator and Global Feature Aggregator, ⊕ indicates a direct summation operation, while $w_{j}^{i}$ represents the weight for the operation j∈{1,2|1,2,3} in the node i∈{3_1,2_1,1_2,2_2,3_2,4_2}, and ϵ is a small value to avoid numerical instability [31].

### 3.4. Embedding Head

Given the fused 2D feature maps from the terminal nodes $P_{1\_2}$, $P_{2\_2}$, $P_{3\_2}$, and $P_{4\_2}$ from a layer of the neck, the embedding head will create the image descriptors, as illustrated in Figure 1 like the GeM head. This head is composed of four mini-heads, one for each terminal node, and similarly to [25,26], we applied a Generalized Mean (GeM) [28] pooling layer with learnable parameters for the pooling process of the 2D feature maps.

Then, given a feature map $P_{i}$ with dimension $C_{in}\times H_{i}\times W_{i}$, where i∈{1_2,2_2,3_2,4_2}, a mini-head produces a vector $f_{i}$ with size $C_{in}$:

(7) $$f_{i}=\mathrm{FC}\left(I_{2}\left(\mathrm{FC}\left(\mathrm{GeM}\left(P_{i}\right)\right)\right)\right),$$

where FC represents a fully connected layer; I_2_, the L2 normalization operator; and GeM, the Generalized Mean pooling layer. The L2 normalization operator is used to apply the Sub-center ArcFace [11] loss during the training process and for minimizing the overall loss [32]. This bottleneck operation is shown in Figure 2c.

The final vector image descriptor $f_{e}$ with size $4\cdot C_{in}$ is composed of the stacking process of the four feature vectors $f_{i}$.

## 4. Experiments

### 4.1. Experimental Setup

#### 4.1.1. Datasets and Evaluation Techniques

The proposed neck model and GeM head for the LGFFEM are trained using three different strategies, using the public ImageNet-1k training dataset [9], which consists of 1,281,167 training images and 1000 object classes. Additionally, we employed the PanNuke [33,34] dataset with the toolbox PathML (http://pathml.org/) (the GNU GPL v2 version of PathML is made available via Open Source licensing), accessed on 10 July 2024, containing 189,744 segmented nuclei and encompassing 19 different types of tissues. Additionally, Kimia Patch24C [12], a training dataset consisting of 22,591 training patches from 24 WSIs representing various tissues, was employed. Image augmentation for PanNuke and Kimia training datasets was carried out using the vision tool Albumentations [35].

The metric evaluation dataset employed for the retrieval process was the Kimia Patch24C test dataset, comprising a total of 1325 patches from 24 WSIs representing various tissues. The metric used was the same as that proposed by [36], defined as

(8) $$\eta_{p}=\frac{\sum_{s\in S}\left|R\cap \Gamma_{s}\right|}{n_{tot}}$$

and

(9) $$\eta_{w}=\frac{1}{24}\sum_{s\in S}\frac{\left|R\cap \Gamma_{s}\right|}{n_{\Gamma_{s}}}$$

Here, $\eta_{p}$ represents the patch-to-scan accuracy, and $\eta_{w}$ represents the whole-scan accuracy within the retrieved image set *R*. The set $\Gamma_{s}=P_{s}^{i}|s\in S,i=1,2,\ldots ,n_{\Gamma_{s}}$ corresponds to the patches associated with scan *s*, where s=0,1,2,…,23, and the total number of patches is $n_{tot}=1325$. The overall accuracy is defined as $\eta_{tot}=\eta_{p}\cdot \eta_{w}$.

**Figure 3:**
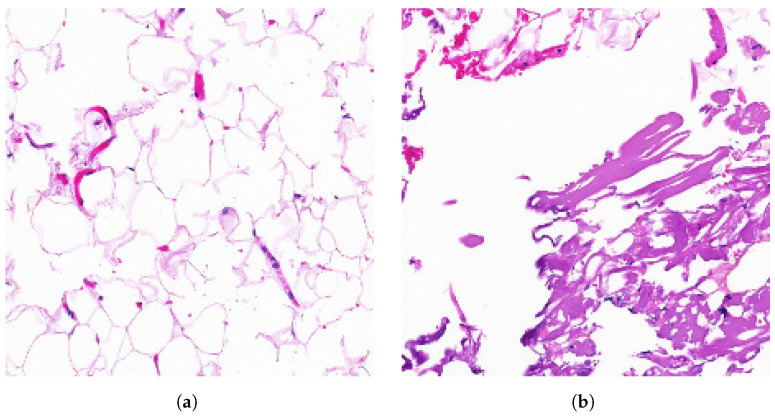
Query image selected for the class set *S*0 and their first retrieve image from the Kimia Patch24C dataset. (**a**) Query image ID S0-1. (**b**) First image retrieved ID S0-2.

While these metrics provide a quantitative evaluation, they do not offer a comprehensive understanding of the neck model’s behavior. To address this, we augment our evaluation by analyzing Class Activation Maps (CAMs) across three neck layers of the trained models, utilizing the Grad-CAM visual explanation technique. In this case, the results aim to visualize the behavior of the feature fusion operations in the outer aggregation fusion nodes $P_{1\_2}$, $P_{2\_2}$, $P_{3\_2}$, and $P_{4\_2}$, as described in Equation (6). We selected the first image from the class set *S*0 of the test dataset of Kimia Patch24C as a query image and retrieved the first result using the FAISS library. These two images, one query image and one retrieved image, are shown in Figure 3.

#### 4.1.2. Backbone and Implementation Detail

We implemented our framework model using PyTorch version 23.05 [37], with the pre-trained backbone ConvNeXt V2 [30]. For the neck model, we used 3 layers of LGFFN, with an inner channel $C_{in}$ size of 512. For the GeM head, we used a vector image descriptor $f_{e}$ size of 2048. The GeM pooling layer of the parameters used are p=4.6 and $\epsilon =1\times 10^{-6}$. As the index retrieval algorithm, we utilized the FAISS library [38] with an Euclidean index. The complete code of the models, along with the configuration files, the trained model’s weights, and examples of training, are available at https://github.com/camilo-nunez/ffnir, accessed on 1 July 2024.

We trained the models using the computer resources NVIDIA RTX 6000 ADA, 64 GB RAM, and AMD Ryzen 9 5950X 16-Core Processor. The neck model with the GeM head model had a total of $1.45\times 10^{7}$ trainable parameters, and the whole training process took around 60 h.

#### 4.1.3. Training Strategies

We conducted our experiments using three different strategies for the training of the neck and the GeM head: (A) training using only ImageNet-1k, (B) training using ImageNet-1k + PanNuke, and (C) training using ImageNet-1k + PanNuke + Kimia Patch24C.

All strategies were trained using the Sub-center ArcFace [11], with parameters m=17.2 and s=64.

The models were optimized using AdamW with a cosine annealing schedule, where the initial learning rate was set at 5 × 10^−3^, and the minimum learning rate was set at 8 × 10^−5^. Strategy A used a batch size of 64 for 60 epochs, while strategies B and C used a batch size of 64 for 300 epochs. All the images were resized to 224×224 pixels.

### 4.2. Results and Analysis

#### 4.2.1. Metric Evaluation

The main objective of training with three different transfer learning techniques is to understand how domain generalization decreases as the specific-domain dataset expands. To achieve this, we present the accuracy results $\eta_{p}$, $\eta_{w}$, and $\eta_{tot}$ for the three training strategies in Table 1, using the test dataset of Kimia Patch24C. As seen, the accuracy metrics improve when the proposed model is trained on different types of image domains. In detail, the domain of classic images from ImageNet-1k shows a lower accuracy compared to training using two specific-domain datasets, PanNuke and Kimia Patch24C. Furthermore, high accuracy is achieved when transferring learning from the first two datasets, ImageNet-1k and PanNuke, in the training process of strategy C for the specific-domain Kimia Patch24C.

For the baseline analysis, we compared our proposed model with the model used in [39] and a more recent model in [14]. The accuracy results of these two baseline models and our proposed model are shown in Table 2. Our model, trained with strategy C, surpassed the accuracy achieved by the two baseline models.

#### 4.2.2. Explanation with CAM

Based on preliminary clinical analysis, the query image represents mature adipose tissue stained with Hematoxylin and Eosin (H&E) and Masson’s trichrome. In the image with ID S0-2 (Figure 3b), mature adipose tissue is visible in the upper left corner, while fibroblastic proliferation is observed in the bottom right corner.

On the other hand, to explore the tissue morphology and cell structure of the image, we applied GradCam++ [40] visual explanation over the outer aggregation fusion nodes $P_{1\_2}$, $P_{2\_2}$, $P_{3\_2}$, and $P_{4\_2}$ from Equation (6). The objective was to understand how the model interprets these characteristics.

**Figure 4:**
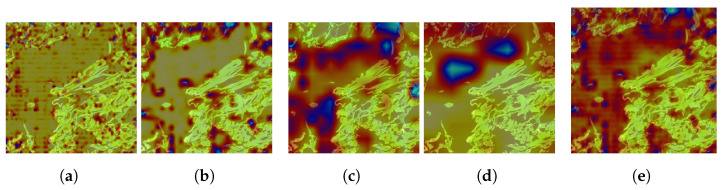
Grad-CAM applied to the first layer of the neck used in strategy C for the first image’s retrieved ID S0-2. (**a**) Grad-CAM applied to the outer aggregation fusion node $P_{1\_2}$ in Layer 1. (**b**) Grad-CAM applied to the outer aggregation fusion node $P_{2\_2}$ in Layer 1. (**c**) Grad-CAM applied to the outer aggregation fusion node $P_{3\_2}$ in Layer 1. (**d**) Grad-CAM applied to the outer aggregation fusion node $P_{4\_2}$ in Layer 1. (**e**) Grad-CAM applied to the collapse of all outer aggregation fusion nodes in Layer 1.

**Figure 5:**
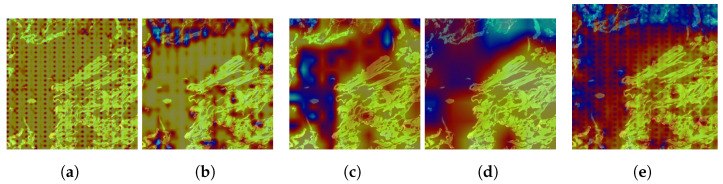
Grad-CAM applied to the second layer of the neck used in strategy C for the first image’s retrieved ID S0-2. (**a**) Grad-CAM applied to the outer aggregation fusion node $P_{1\_2}$ in Layer 2. (**b**) Grad-CAM applied to the outer aggregation fusion node $P_{2\_2}$ in Layer 2. (**c**) Grad-CAM applied to the outer aggregation fusion node $P_{3\_2}$ in Layer 2. (**d**) Grad-CAM applied to the outer aggregation fusion node $P_{4\_2}$ in Layer 2. (**e**) Grad-CAM applied to the collapse of all outer aggregation fusion nodes in Layer 2.

**Figure 6:**
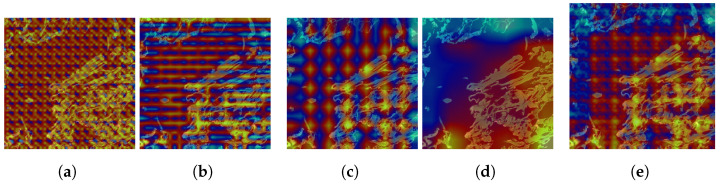
Grad-CAM applied to the third layer of the neck used in strategy C for the first image’s retrieved ID S0-2. (**a**) Grad-CAM applied to the outer aggregation fusion node $P_{1\_2}$ in Layer 3. (**b**) Grad-CAM applied to the outer aggregation fusion node $P_{2\_2}$ in Layer 3. (**c**) Grad-CAM applied to the outer aggregation fusion node $P_{3\_2}$ in Layer 3. (**d**) Grad-CAM applied to the outer aggregation fusion node $P_{4\_2}$ in Layer 3. (**e**) Grad-CAM applied to the collapse of all outer aggregation fusion nodes in Layer 3.

The results of these visualizations are systematically represented in Figure 4, Figure 5 and Figure 6 for the first retrieved image (ID S0-2), corresponding to the three layers of the neck model.

In the first layer, nodes $P_{1\_2}$ and $P_{2\_2}$ do not identify any morphology or structural type in the image. Despite this, nodes $P_{3\_2}$ and $P_{4\_2}$ recognize some parts of the tissue, as indicated by the red gradient areas, while avoiding the immunohistochemical zones, shown as blue gradient areas. However, they still do not identify any specific morphology or structure. The combined results of the four nodes confirm this behavior, indicating that the initial layer is not effective in capturing the relevant tissue characteristics.

In the second layer, the behavior of nodes $P_{1\_2}$ and $P_{2\_2}$ remains consistent, as they again fail to identify the morphology or structure type in the image. These nodes only display the initialization of the fusion feature in random areas, such as the texture points in node $P_{1\_2}$. This suggests that these nodes are not yet tuned to detect meaningful features in the tissue. However, node $P_{3\_2}$ shows a significant improvement, identifying mature adipose tissue in the upper left corner and the morphology of fibroblastic areas in the bottom right corner. Additionally, this node effectively avoids the immunohistochemical zones, indicating a more refined feature detection capability. Node $P_{4\_2}$, on the other hand, loses its focus on the mature adipose tissue but maintains its attention on the fibroblast area. The combined results show that node $P_{4\_2}$ has a greater influence than the other three nodes in this layer, suggesting that it may play a dominant role in feature detection at this stage.

In the third layer, nodes $P_{1\_2}$, $P_{2\_2}$, and $P_{3\_2}$ exhibit delayed and sluggish fusion behavior. This behavior indicates that these nodes are not effectively integrating the detected features into a coherent representation. Only node $P_{4\_2}$ focuses on the morphology zone, particularly the fibroblast area, demonstrating a more targeted and effective feature detection. The combined results from this layer display the delayed and sluggish fusion behavior across all nodes, emphasizing the challenges in achieving effective feature integration. This suggests that further refinement and tuning of the model are needed to improve its ability to accurately identify and integrate tissue characteristics across multiple layers.

#### 4.2.3. Visualization of Learned Embeddings

**Figure 7:**
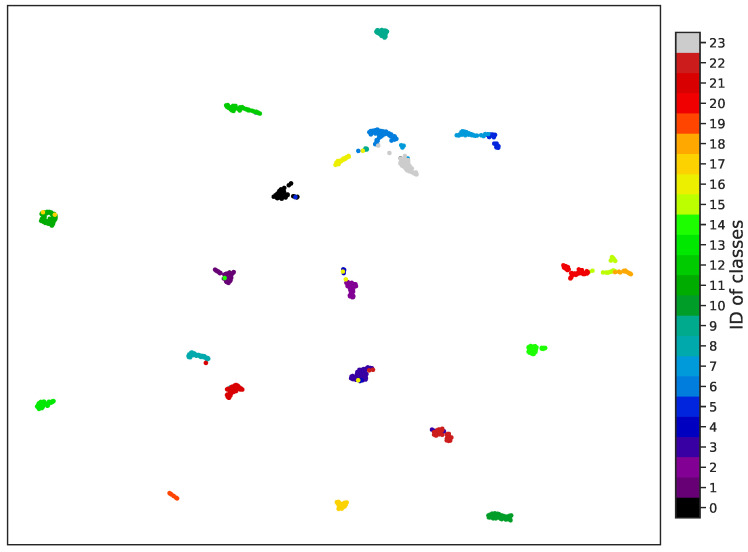
Visualization of the 2D projection of embeddings from the Kimia Patch24C test dataset using strategy C. Each dot represents an image, and each color represents a class of tissue.

As support for our results, we show a projection in a 2D space of the embeddings retrieved by our model in Figure 7. This figure shows the clusters associated with the 24 different classes of tissues in the test dataset.

## 5. Conclusions

We designed and implemented a unified model framework for extracting embeddings of image descriptors from histopathological images using multi-scale local–global fused features. The results achieved by the LGFFEM indicate that the proposed method can surpass baseline models with a new training strategy and multi-scale local–global fused features. The proposed method is designed with fewer parameters compared to classical pre-trained models, allowing it to be trained on more specific-domain medical images without the necessity of using complex computer resources.

In particular, the LGFFEM shows promising results in addressing important study questions, such as how specific-domain training affects the accuracy of the model, how tissue morphology and cell structure can be interpreted by local–global feature fusion, and how the generation of visual descriptor embeddings can preserve high-order image semantic structures. Further studies in other specific-domain areas, such as X-ray or pap smear images, are warranted to explore the capability of this model in order to create a comprehensive web-based CBMIR for multiple-medical-imaging specialists.

## Figures and Tables

**Table 1:** Retrieve accuracy (%) for strategies A, B and C.

Strategy	Pre-Training Data	$\eta_{p}$	$\eta_{w}$	$\eta_{\mathrm{tot}}$
A	IN-1K	72.08	74.37	53.6
B	IN-1K + PanNuke	77.36	79.28	61.33
C	IN-1K + PanNuke + Kimia	99.40	99.47	98.87

**Table 2:** Retrieve accuracy (%) for baselines models and the best model obtained from strategy C.

Method	$\eta_{p}$	$\eta_{w}$	$\eta_{\mathrm{tot}}$
DenseNet 121 [12]	95.92	95.51	91.62
MA + MS-loss [14]	97.89	97.00	94.95
LGFFEM [ours]	99.40	99.47	98.87

## Data Availability

Data are contained within the article.
